# The patient’s voice: a cross-sectional study of physical health and disability in juvenile idiopathic arthritis

**DOI:** 10.1186/s12969-024-01034-7

**Published:** 2024-11-18

**Authors:** Sofie Mikalsen Arneng, Isabelle Pignatel Jenssen, Anette Lundestad, Lena Cetrelli, Oskar Angenete, Ellen Nordal, Karin B. Tylleskär, Pål Richard Romundstad, Marite Rygg

**Affiliations:** 1 Department of Clinical and Molecular Medicine, Faculty of Medicine and Health Sciences (IKOM), Norwegian University of Science and Technology (NTNU), Trondheim, Norway; 2 Department of Pediatrics, St. Olavs Hospital, Trondheim University Hospital, Trondheim, Norway; 3 Center for Oral Health Services and Research, Mid-Norway (TkMidt), Trondheim, Norway; 4 The Public Dental Health Service, Trøndelag County, Trondheim, Norway; 5 Department of Radiology and Nuclear Medicine, St. Olavs Hospital, Trondheim University Hospital, Trondheim, Norway; 6 Department of Circulation and Medical Imaging, Faculty of Medicine and Health Sciences, Norwegian University of Science and Technology, Trondheim, Norway; 7 Department of Clinical Medicine, The Arctic University of Norway (UiT), Tromsø, Norway; 8 Department of Pediatrics, University Hospital of North Norway (UNN), Tromsø, Norway; 9 Child and Youth Clinic, Haukeland University Hospital, Bergen, Norway; 10 Department of Public Health and Nursing, Faculty of Medicine and Health Sciences, Norwegian University of Science and Technology (NTNU), Trondheim, Norway

**Keywords:** Juvenile idiopathic arthritis, Patient-reported outcome measures, PROM, Physical disability, Physical health, Psychosocial health, Self-reported, Quality of life, Cross-sectional study

## Abstract

**Background:**

With increasing focus on patient-reported outcome measures (PROMs) in chronic rheumatic diseases, we aimed to evaluate the self-reported physical and psychosocial health in children with juvenile idiopathic arthritis (JIA) compared to matched population-based controls. Furthermore, we aimed to study the association of patient- and physician-reported outcome measures in JIA with patient-reported physical disability.

**Methods:**

We used data from a Norwegian JIA cohort study (NorJIA), including clinical characteristics and outcome measures in participants with JIA and sex- and age-matched population-based controls. Self-reported physical and psychosocial health were assessed using the generic Child Health Questionnaire (CHQ). Comparisons between children with JIA and controls were performed by test of proportions for categorical variables and t-test for continuous variables. To evaluate the association of patient- and physician-reported outcome measures with patient-reported physical disability, assessed with the Child Health Assessment Questionnaire (CHAQ) in children with JIA, we used logistic regression to estimate adjusted odds ratio (OR) with 95% confidence interval (CI).

**Results:**

In total, 221 participants with JIA (59.3% females, median age 12.7 years) and 207 controls with available data were included. In the JIA group, 24.3% scored below the norm for physical health (CHQ PhS < 40) and 8.7% scored below the norm for psychosocial health (CHQ PsS < 40). The corresponding numbers for the control group were 0.5% and 1.9%, respectively. In the JIA group, 57.9% reported physical disability (CHAQ > 0). Several patient-reported outcome measures, such as poor physical health (CHQ PhS < 40), disease-related pain, and the patient’s global assessment of disease impact on wellbeing, were strongly associated with self-reported physical disability (CHAQ > 0), adjusted OR 19.0 (95% CI 5.6, 64.1), 14.1 (95% CI 6.8, 29.2), and 14.0 (95% CI 6.2, 31.6), respectively.

Associations were also found for active disease according to Wallace (adjusted OR 36.3, 95% CI 10.3, 128.1), and physician-reported global assessment of disease activity (adjusted OR 6.2, 95% CI 3.1, 12.6).

**Conclusions:**

The strong association between patient- and physician-reported outcome measures and patient-reported physical disability strengthens the importance of including the patient’s voice in a comprehensive evaluation of patient outcome in JIA.

**Trial registration:**

ClinicalTrials.gov (No: NCT03904459).

**Supplementary Information:**

The online version contains supplementary material available at 10.1186/s12969-024-01034-7.

## Background

Juvenile idiopathic arthritis (JIA) is one of the most common conditions causing chronic arthritis in children [1] with a particular high incidence in the Nordic countries [2, 3]. JIA is a complex disease with heterogenous clinical characteristics [4]. Children living with JIA face an unpredictable disease course with periods of exacerbations and remissions. A follow-up study conducted in the Nordic countries found that only 33% of patients were considered as being in clinical remission off medication 18 years after disease onset [5]. With the development of new and more effective drugs in JIA during the later years, the ultimate treatment goal is no longer only to reduce suffering, but to achieve sustained inactive disease (remission) [6]. In order to reach this ultimate goal, several aspects of the disease must be considered. As the various clinical features and consequences of the disease may be assessed and weighted differently by the physician, parents, and patient [7], there is a need to include the patient’s voice in the disease assessment.

Over the past decade, patient-reported outcome measures (PROMs) have been given increasing attention and significance in the clinical evaluation and follow-up of JIA, as well as in JIA research [8]. PROMs are thought to help improve the patient’s treatment by providing the physician with important insight into both the patient’s perception of their condition as well as what the patient considers to be the most pressing issues [8]. This insight may be used to tailor the individual patient’s care and treatment. Thus, PROMs are important instruments in the effort to enhance the patient’s quality of care [8–10]. In 1997, Paediatric Rheumatology INternational Trials Organisation (PRINTO) defined a core set of outcome measures to be used in clinical trials. In 2001, the Childhood Health Assessment Questionnaire (CHAQ) was selected as the principal disease-specific tool to assess the child’s physical disability [11]. Another PROM commonly used to assess JIA, is the Child Health Questionnaire (CHQ) [11]. The CHQ is a multidimensional tool used for measuring the physical and psychosocial health of the child unrelated to any specific disease [12]. The generic nature of the CHQ allows comparison with control groups. Other examples of PROMs in JIA are patient-reported pain, morning stiffness, and global assessment of disease impact on well-being. Despite the increased focus, limited research has been done on the associations between CHAQ, CHQ and other PROMs, and especially on the associations of CHAQ with physician-reported outcome measures and composite disease activity measures.

The aim of this study was to study patient-reported physical health – assessed with the generic Physical Summary Score of The Child Health Questionnaire (CHQ PhS) – in a cohort of Norwegian children with JIA compared to a matched population-based control cohort. Furthermore, within the JIA group, we wanted to evaluate the association between several patient- and physician-reported disease activity and outcome measures and patient-reported physical disability, assessed with the disease-specific CHAQ.

## Methods

### Study design

The NorJIA study is a prospective, multicenter, observational study, https://norjia.com/. Cases were recruited from out-patient clinics at the pediatric departments of St. Olavs University Hospital in Trondheim, Mid-Norway, Haukeland University Hospital in Bergen, Western Norway, and University Hospital of North Norway in Tromsø, Northern Norway. The participants with JIA, who had random disease durations, were examined twice, two years apart (± 3 months). The baseline clinical examinations were set between 2015 and 2018. Inclusion criteria were children between the age of 4 and 16 years meeting the International League of Associations for Rheumatology (ILAR) criteria for JIA [13], with parents’ or legal guardians’ (in this article collectively referred to as *parents*) informed consent. The lower cut-off of 4 years was determined as the lowest age we could ask a child and his/her parents to participate in a comprehensive study consisting of two consecutive study days with various examinations. There were no exclusion criteria.

The matched population-based control cohort consisted of children without JIA, matched 1:1 for sex and age. They were recruited from dental offices related to the Center for Oral Health Services and Research, Mid-Norway (TkMidt), the Oral Health Center of Expertise in Western Norway (TkV), and the Public Dental Health Service Competence Center of Northern Norway (TkNN). The children were called in for a free dentist appointment according to the Norwegian public dental service policy and could therefore be considered a random sample from the general population. The study design of this study is cross-sectional, using data from the baseline examination. For this particular study, the inclusion criteria were participation in the baseline visit of the NorJIA study with a completed CHAQ. In sub-analyses, participants, both those with JIA and controls, with a completed CHQ were included.

### Data collection

The NorJIA study included extensive clinical, laboratory, radiological, and oral examinations at baseline and after two years’ follow-up. It also included several PROMs, including CHAQ and CHQ. Relevant data from the baseline study visit included: Sex, age at disease onset and at study visit, parental education level, ethnicity, anthropometric data, blood test results, JIA category according to the ILAR classification criteria [13], joint status evaluated by the physician at the study visit (including number of active joints), disease status at the study visit according to Wallace and the American College of Rheumatology (ACR) criteria [14, 15], and medication (past and present). Erythrocyte sedimentation rate (ESR) and C-reactive protein (CRP) were assessed at the clinical examination. Other blood tests such as human leukocyte antigen B27 (HLA-B27), rheumatoid factor (RF), and anti-nuclear antibody (ANA) were all measured around the time of the diagnosis and registered in the NorJIA database. RF and ANA were measured twice at least three months apart; for ANA an indirect immunofluorescence assay on HEp-2 cells was used. In addition to the clinical examination, either the patient (if the patient was nine years or older), or the parents (if the child was younger than nine years), filled out the CHAQ form. In addition, pain as well as overall well-being, were reported by the participants (patient or parents, as above) on a visual analogue scale (VAS). They also reported duration of morning stiffness. Parents of both participants with JIA and sex- and age-matched controls (regardless of the patient’s age) filled out the CHQ.

### Measures

Parental education level was reported by the parents of both children with JIA and controls, and subsequently divided into four levels: Primary and middle school (7 to 10 years of education), high school (11 to 13 or 14 years), less than five years of university education, and five or more years of university education. The last two levels will be collectively referred to as *parents with higher education*. The grouping was defined according to the parent with the highest education level.

Body mass index (BMI) was calculated using the following formula: Weight (kilograms)/[height (metres)]^2^ and subsequently adjusted for sex and age according to the International Obesity Task Force (IOTF) cut-off values to provide an Iso-BMI stratification that allows for comparison with adult BMI groups: Underweight (BMI < 18.5), normal (BMI ≥ 18.5 and < 25), overweight (BMI ≥ 25 and < 30), obesity (BMI ≥ 30) [16, 17].

### Patient-reported outcome measures

Patient-reported physical disability was assessed with CHAQ. This questionnaire evaluates the patient’s everyday physical functioning during the previous week, through 30 items divided into eight domains: Dressing, arising, eating, walking, hygiene, reach, grip, and activities. The need for assistance in the form of aids, devices, and help from another person for physical functioning are also registered. According to their questionnaire responses, a global CHAQ score was calculated for each patient. This score ranges from zero (no or minimal physical disability) to three (very severe physical disability) [11, 18]. The CHAQ is cross-culturally validated and translated into many languages, including Norwegian [19]. Patient/parent-reported disease-related pain during the last week was measured on a 21-numbered circle VAS, where zero equals “no pain at all” and 10 equals “very severe pain”. The VAS pain score is extensively used both in clinical follow-up and in research, both in the form of a continuous 10 cm scale and a 21-numbered scale [20]. The patient’s global assessment of disease impact on overall well-being (PatGA) is a tool where the patient/parent scores the influence of the disease on the patient’s everyday life during the last week on a 21-numbered circle VAS from 0–10, where zero represents “no influence at all” and 10 “severely influenced”. This tool is extensively used, both in clinical follow-up of JIA and in research [10, 20]. Morning stiffness during the last week was reported by the patient (≥ 9 years old) or parents (for children < 9 years old) in minutes. The generic 50-item Child Health Questionnaire-Parent Version (CHQ-PF50) – in this article referred to as *CHQ* – includes questions about topics such as general health, physical functioning, physical pain, mental health, as well as the impact on both the patient, parents, and family in general, reported for the last four weeks [11, 12]. The responses were compiled into a summarized score with a scale from 1 to 100, where higher scores indicate a higher level of functioning and well-being comparable to norm scores (50 ± 10) from the general U.S. population. The responses are used to calculate a Physical Summary Score (PhS) and a Psychosocial Summary Score (PsS), also with norm scores (50 ± 10).

### Physician-reported outcome measures

The physician’s global assessment of disease activity (PhysGA) was reported by the physician on a 21-numbered circle VAS from zero to 10 where zero equals “no activity” and 10 equals “high activity” [20].

### Composite disease activity measures

Disease status was defined according to Wallace et al. [14] with modifications according to the ACR endorsed criteria [15]. Inactive disease was accordingly defined as no active arthritis or uveitis, no systemic manifestations of JIA, normal acute inflammatory markers (ESR or CRP) or, if elevated, not attributable to JIA, physician’s evaluation indicating no activity (PhysGA = 0), and morning stiffness < 15 min.

### Medication

This study focused on the patients’ past and current use of disease-modifying antirheumatic drugs (DMARDs), including synthetic DMARDs (sDMARDs) and/or biologic DMARDs (bDMARDs). Synthetic DMARDs included methotrexate, hydroxychloroquine, cyclosporine, and mycophenolate mofetil. Biologic DMARDs included etanercept, infliximab, adalimumab, tocilizumab, abatacept, certolizumab, golimumab, and rituximab.

### Statistical analysis

To describe the clinical characteristics and disease activity of the cohort, either mean and standard deviation (SD) or median and 1st to 3rd interquartile ranges (IQR) were used for continuous variables. Absolute frequencies and percentages were used for categorical variables. For proportions, we estimated percent point differences with 95% confidence intervals using the prtest command in STATA.

To study patient-reported physical health in the JIA cohort compared to the matched control cohort, the mean Physical Summary Score (CHQ PhS) and the mean Psychosocial Summary Scores of the Child Health Questionnaire (CHQ PsS) of the JIA cohort was compared to the control cohort using t-test for independent samples. CHQ scores were also dichotomized into normal (CHQ ≥ 40) and poor (CHQ < 40) physical or psychosocial health.

To evaluate the association of patient- and physician-reported outcome measures with patient-reported physical disability (CHAQ > 0), we used logistic regression analyses and adjusted for sex, age, disease duration, and Iso-BMI chosen by *a priory* knowledge, to estimate the adjusted odds ratio (OR) with 95% confidence interval (CI). The CHAQ scores were dichotomized into 0 (no disability) or > 0 (disability) and used as the main outcome variable. The other variables were categorized as follows; VAS pain (0 = no pain, > 0 = pain), PatGA (0 = no disease influence on well-being, > 0 = disease influence on well-being), morning stiffness (< 15 min = no substantial morning stiffness, ≥ 15 min = substantial morning stiffness), PhysGA (0 = no disease activity, > 0 = disease activity), and past and present medication (no DMARDs, DMARDs). We categorized disease status according to Wallace et al. [14, 15] into three condensed levels; remission off medication, inactive disease, and active disease. Remission off medication = remission off any antiarthritic or anti-uveitis medication for ≥ 12 continuous months. Inactive disease = inactive disease on medication < 6 months or off medication < 12 months, or remission on medication (inactive disease on medication for more than six months). Active disease = flare or continuous active disease. JIA categories were dichotomized to oligoarticular persistent JIA or all other JIA categories. Statistical analyses were carried out using STATA version 16, software (STATA Corp., College Station, Texas, USA).

## Results

### Sample characteristics

**Fig. 1:**
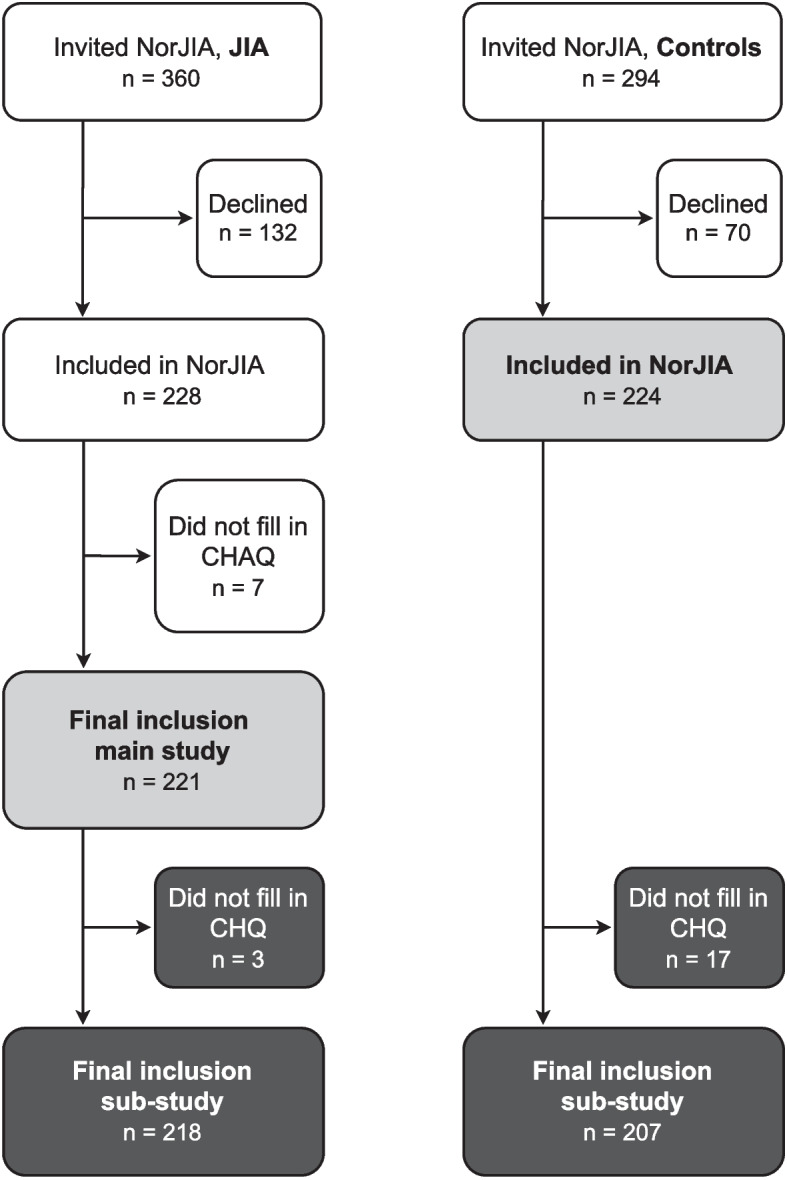
Flow chart of children and adolescents with juvenile idiopathic arthritis (JIA) and controls in the study. Abbreviations: NorJIA = the Nordic Juvenile Idiopathic Arthritis study, JIA = juvenile idiopathic arthritis, CHAQ = Child Health Assessment Questionnaire, CHQ = Child Health Questionnaire (parent form)

Of the 360 children with JIA that were invited to participate in the NorJIA study, 228 accepted, yielding a response rate of 63% (Fig. 1). Of the 228 participants, seven (three females and four males) were excluded from the final study population, as their CHAQ forms were lacking or missing responses to one or more sections. Five of the remaining participants had missing responses to one or two of the CHAQ questions; these were kept in the study. Thus, 221 participants with JIA were included in the final study population. Of these 221, three (two females, one male) did not complete the CHQ, leaving 218 participants with JIA for comparison with the control group in the sub-study on self-reported physical health. The response rate among the controls was 224 of 294 invited controls (76%), and 17 of these (eleven females and six males) did not complete the CHQ, resulting in 207 controls in the sub-study. Both children with JIA and potential controls were invited once, and we did not try to further increase the response rate. Of the 132 patients with JIA who declined to participate in the study, 58% were female compared to 59% in the included group. The mean age among those who declined tended to be lower than the included (11 years, SD 3.5 versus 12 years, SD 3.2). No substantial differences in clinical characteristics were found comparing the children who were not included in the sub-study with the included children (results not shown).

### Demographics

In the study population, 59.3% of participants with JIA were female, median age was 12.7 years, with a similar age and sex distribution in the control group (Table 1). The proportion of parents with higher education was 11.2 percent points (95% CI 0.0%, 19.3%) higher in the control group than in the JIA group. The majority, 70.1% in the JIA group and 75.1% in the control group, were classified with normal weight. The proportions of both underweight and obese participants tended to be slightly higher in the JIA group compared to the control group, by 2.6 percent points (95% CI -1.6%, 6.9%) and 2.2 percent points (95% CI -1.4%, 5.8%), respectively.

**Table 1:** Clinical characteristics of the study population

	**JIA**		**Controls**	
	***N***^***a***^	***Values***	***N***^***a***^	***Values***
Females, n (%)	221	131 (59.3)	224	134 (59.8)
Age at examination, median years (IQR)	221	12.7 (9.4, 14.7)	224	12.6 (9.6, 14.9)
Parent with higher education^b^, n (%)	212	148 (69.8)	216	175 (81.0)
Caucasian ethnicity^c^, n (%)	220	214 (97.3)	221	205 (92.8)
Iso-BMI^d^, n (%)				
*Underweight*	221	15 (6.8)	217	9 (4.1)
*Normal weight*	221	155 (70.1)	217	163 (75.1)
*Overweight*	221	40 (18.1)	217	39 (18.0)
*Obesity*	221	11 (5.0)	217	6 (2.8)

*N* Number, *JIA* Juvenile Idiopathic Arthritis, *IQR* Interquartile range, *Iso-BMI* Body Mass Index adjusted for age and sex

^a^Number of participants assessed for each variable, excluding missing/unknown values. Total JIA group, *N* = 221. Total control group, *N* = 224

^b^Parent education was divided into 4 levels: Primary and middle school (7 to 10 years of education), high school (11 to 13 or 14 years), university education < 5 years, and university education > 5 years; the last 2 levels were grouped together as parents with higher education. The grouping was defined according to the parent with the highest education level

^c^Ethnicity was divided into two groups: Caucasian and non-Caucasian according to self-report

^d^Calculated using the formula: Weight (kilograms)/[height (metres)]^2^ and subsequently adjusted for age and sex according to The International Obesity Task Force (IOTF) cut-off values to allow for comparison with adult BMI groups as defined by Cole TJ, et al.: Underweight: < 18.5, normal weight: 18.5–24.9, overweight: 25–29.9, obesity: ≥ 30 kg/m^2^

### Disease characteristics in the JIA group

Median age of disease onset in the JIA group was 6.1 years, and median disease duration was 4.6 years (Table 2). At the study visit, 21.7% had one or more active joints, and 65.2% received ongoing treatment with DMARDs (including 38.9% with bDMARDs). The physician assessed 33.9% of the children as having active disease (PhysGA VAS > 0). Disease-related pain (VAS pain > 0) was reported by 62.9% of the children with JIA.

**Table 2:** Disease characteristics of the juvenile idiopathic arthritis (JIA) group assessed at the study visit

	***N***^***a***^	***Values***
Age at disease onset, median years (IQR)	221	6.1 (2.3, 10.4)
Disease duration, median years (IQR)	221	4.6 (2.6, 8.2)
HLA-B27 positive, n (%)	221	62 (28.1)
RF positive, n (%)	221	6 (2.7)
ANA positive, n (%)	199	56 (28.1)
Uveitis^b^, n (%)	220	28 (12.7)
JIA category, n (%)^c^		
*Oligoarticular persistent*	221	76 (34.4)
*Oligoarticular extended*	221	23 (10.4)
*Polyarticular RF negative*	221	51 (23.1)
*Polyarticular RF positive*	221	4 (1.8)
*Enthesitis-related arthritis*	221	22 (10.0)
*Psoriatic arthritis*	221	9 (4.1)
*Systemic*	221	7 (3.2)
*Undifferentiated*	221	29 (13.1)
ESR ≥ 20, n (%)	218	8 (3.7)
CRP ≥ 5, n (%)	217	12 (5.5)
Children with active joints > 0, n (%)	221	48 (21.7)
Disease status^d^, n (%)		
*Remission off medication*	221	29 (13.1)
*Inactive*	221	106 (48.0)
*Active*	221	86 (39.0)
Ongoing DMARDs^e^, n (%)	221	144 (65.2)
PhysGA > 0, n (%)	221	75 (33.9)
VAS pain > 0, n (%)	221	139 (62.9)
CHAQ score > 0, n (%)	221	128 (57.9)

*N* Number, *JIA* Juvenile idiopathic arthritis, *IQR* Inter-quartile range, *HLA-B27* Human leukocyte antigen B27, *RF* Rheumatoid factor, assessed twice at least 3 months apart, *ANA* Anti-nuclear antibody, assessed twice at least 3 months apart using HEp-2 cells, *ESR* Erythrocyte sedimentation rate, *CRP* C-reactive protein, *DMARDs* disease modifying anti-rheumatic drugs, *VAS pain* Visual analogue scale for self-reported pain (0 = No pain, 10 = maximal pain) assessed by the patient/parent on a 21-numbered circle VAS, *PhysGA* Physician's global assessment of disease activity (0 = No activity, 10 = high activity) assessed by the physician on a 21-numbered circle VAS, *CHAQ* Childhood Health Assessment Questionnaire (0 = no disability, 3 = maximal disability) assessed by the patient/parent

^a^N assessed for each variable, excluding missing/unknown values. Total participants, *N* = 221

^b^Treated for uveitis at any time during the disease course

^c^Categories defined according to the International League of Association for Rheumatology (ILAR) classification criteria

^d^Disease status according to [14] and [15]: Remission off medication = inactive disease off medication for ≥ 12 months. Inactive disease = inactive disease on medication < 6 months or off medication < 12 months, or remission on medication (inactive disease on medication for > 6 months). Active disease = flare or continuous active disease

^e^Ongoing refers to ongoing DMARDs for arthritis or uveitis at the study visit, including synthetic DMARDs (methotrexate, hydroxychloroquine, cyclosporine, mycophenolate mofetil) and/or biologic DMARDs (etanercept, infliximab, adalimumab, tocilizumab, abatacept, certolizumab, golimumab, rituximab)

### Parent-reported physical and psychosocial health – CHQ

Children with JIA reported lower mean physical health than the controls, with a mean physical summary score (CHQ PhS) of 45.7 in JIA versus 56.0 in controls. The corresponding mean difference was 10.3 (95% CI 8.7, 11.9) (Table 3). Likewise, 24.3% of the JIA group reported poor physical health with a physical summary score below the cut-off for the norm score (< 40), compared to 0.5% of the control group, with a corresponding percent point difference of 23.8% (95% CI 18.1%, 29.6%).

**Table 3:** Self-reported physical and psychosocial health in participants with juvenile idiopathic arthritis (JIA) and controls

	***JIA*** *N*^*a*^* = 218*	***Controls*** *N*^*a*^* = 207*	***Difference***	***95% CI***
Mean Physical Summary Score (CHQ PhS)				
*Mean (SD)*	45.7 (11.0)	56.0 (4.1)	10.3^b^	8.7, 11.9
*n* < *40 (%)*	53 (24.3)	1 (0.5)	23.8^c^	18.1, 29.6
Mean Psychosocial Summary Score (CHQ PsS)				
*Mean (SD)*	52.9 (8.1)	55.4 (6.4)	2.5^b^	1.1, 3.9
*n* < *40 (%)*	19 (8.7)	4 (1.9)	6.8^c^	2.6, 11.0

*JIA* Juvenile idiopathic arthritis, *CI* Confidence interval, *CHQ* Child Health Questionnaire as assessed by the 50-item Child Health Questionnaire-Parent Version (CHQ-PF50), *PhS* Physical Summary Score, *N* Number, *SD* Standard deviation, *PsS* Psychosocial Summary Score

^a^Number of participants assessed, excluding missing/unknown values. Total participants, *N* = 218, excluding 3 participants with missing CHQ (2 females, 1 male). Total controls, *N* = 207, excluding 17 participants with missing CHQ (11 females, 6 males)

^b^Difference in means, according to T-test

^c^Difference in proportions, according to PR-test

The difference in reported psychosocial health was less pronounced, with mean psychosocial summary scores (CHQ PsS) 2.5 percent points (95% CI 1.1%, 3.9%) lower in the JIA group than in controls (Table 3). The proportion of children reporting poor psychosocial health (CHQ PsS < 40) was 6.8 percent point higher in JIA compared to controls (95% CI 2.6%, 11.0%). Overall, no substantial differences were found between the CHQ scores of females and males in the JIA group (Additional file 1). Likewise, no substantial differences were seen according to age (OR 1.0, 95% CI 0.9, 1.1 per year for ChQ PhS < 40, OR 1.1, 95% CI 0.9, 1.2 per year for ChQ PsS).

### Self-reported physical disability according to sociodemographics, disease duration, and JIA categories

**Fig. 2:**
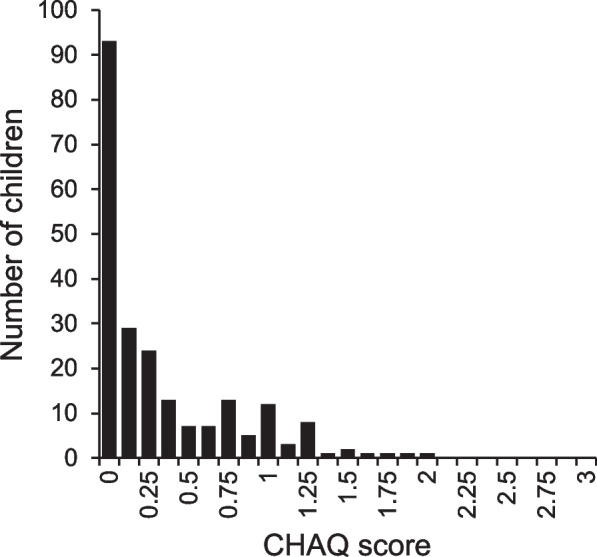
Distribution of The Childhood Health Assessment Questionnaire (CHAQ) scores (range 0–3, 0 = no disability, 3 = maximum disability) reported by the children in the juvenile idiopathic arthritis (JIA) group (*n* = 221). In children less than 9 years (*n* = 45), the parents answered the questionnaire on behalf of their children

More than half (57.9%) of the children with JIA reported some disease-related physical disability (CHAQ > 0) (Table 2). The children with JIA tended to report a disability score between zero and one (mean score of 0.3), and none of the participants scored more than two (Fig. 2).

Females tended to report more physical disability than males (Additional file 2). The proportion of females with JIA reporting physical disability (CHAQ > 0) was 64.1%, and 15.2 percent points (95% CI 2.0%, 28.4%) higher than in males (48.9%) (Table 4). Among participants with JIA below nine years, whose parents filled out the CHAQ forms and pain scores, physical disability tended to be reported slightly higher compared to participants nine years or older, who filled out the CHAQ forms and pain scores themselves (percent point difference 6.5%, 95% CI –9.3%, 22.3%). Those with shorter disease duration (below five years) tended to report slightly higher physical disability compared to those with longer disease duration (above five years), percent point difference 13.4% (95% CI 0.4%, 26.5%). Children with obesity or overweight tended to report more physical disability compared to those with normal weight (percent point difference 14.5%, 95% CI –0.3%, 29.2%). No clear pattern was seen between the different JIA categories and self-reported physical disability, pain, physician-reported global disease activity, and remission status. Females tended to report disease-related pain (VAS pain > 0) more often than males, with 8.6 percent point (95% CI -4.3%, 21.7%) difference. Sex differences were less evident for physician-reported global assessment of disease activity (PhysGA).

**Table 4:** Disease characteristics according to sex, age, and disease categories in the JIA group

	***N***^***a***^	***CHAQ *> *0***	***VAS pain *> *0***	***PhysGA *> *0***	***Not in remission***^***b***^
Sex, n (%)					
Female	131	84 (64.1)	87 (66.4)	47 (35.9)	118 (90.1)
Male	90	44 (48.9)	52 (57.8)	28 (31.1)	74 (82.2)
Age group, n (%)					
< 9 years	46	29 (63.0)	22 (47.8)	12 (26.1)	42 (91.3)
≥ 9 years	175	99 (56.6)	117 (66.9)	63 (36.0)	150 (85.7)
Disease duration, n (%)					
< 5 years	122	78 (63.9)	86 (70.5)	48 (39.3)	110 (90.2)
≥ 5 years	99	50 (50.5)	53 (53.5)	27 (27.3)	82 (82.8)
Iso-BMI^c^, n (%)					
Underweight	15	5 (33.3)	9 (60.0)	7 (46.7)	12 (80.0)
Normal	155	87 (56.1)	96 (61.9)	53 (34.2)	136 (87.7)
Overweight/obese	51	36 (70.6)	34 (66.7)	15 (29.4)	44 (86.3)
JIA category^d^					
Oligoarticular persistent	76	36 (47.4)	46 (60.5)	23 (30.3)	56 (73.7)
Oligoarticular extended	23	16 (69.6)	14 (60.9)	8 (34.8)	22 (95.7)
Polyarticular RF negative	51	33 (64.7)	34 (66.7)	20 (39.2)	50 (98.0)
Polyarticular RF positive	4	4 (100.0)	4 (100.0)	1 (25.0)	4 (100)
Enthesitis-related arthritis	22	12 (54.6)	13 (59.1)	10 (45.5)	20 (90.9)
Psoriatic arthritis	9	7 (77.8)	7 (77.8)	2 (22.2)	7 (77.8)
Systemic	7	2 (28.6)	3 (42.9)	0 (0.0)	5 (71.4)
Undifferentiated	29	18 (62.1)	18 (62.1)	11 (37.9)	28 (96.6)

*Abbreviations:* *N* Number, *CHAQ* Childhood Health Assessment Questionnaire (0 = no physical disability, 3 = maximal physical disability), *VAS pain* Visual analogue scale for self-reported pain (0 = No pain, 10 = maximal pain) reported by the patient/parent on a 21-numbered circle VAS, *PhysGA* Physician's global assessment of disease activity (0 = No activity, 10 = high activity) reported by the physician on a 21-numbered circle VAS, *RF* Rheumatoid factor

^a^Number of participants assessed for each variable, excluding missing/unknown values. Total participants, *N* = 221

^b^Not in remission = Patients who do not fit the criteria for “Remission off medication” (inactive disease off medication for >12 months) as defined by Wallace et al.

^c^Calculated using the formula: Weight (kilograms)/[height (metres)]^2^ and subsequently adjusted for age and sex according to The International Obesity Task Force (IOTF) cut-off values to allow for comparison with adult BMI groups as defined by Cole TJ, et al.: Underweight: <18.5, normal weight: 18.5-24.9, overweight/obese: ≥25 kg/m^2^

^d^Categories defined according to the International League of Association for Rheumatology (ILAR) classification criteria

### Association between disease outcomes and self-reported physical disability (CHAQ)

Disease outcome measures, including other patient-reported measures, physician-reported measures, and medication, was compared to self-reported disease-related physical disability (CHAQ scores) (Table 5). Of children reporting some degree of disease-related pain, 78.4% also reported physical disability (CHAQ > 0). On the other hand, among those reporting no pain, only 23.2% reported physical disability. Among children with JIA reporting poor physical health (CHQ PhS score below 40), 94.3% also reported physical disability. When the physician reported ongoing disease activity (PhysGA > 0), 82.7% of the children reported physical disability. However, even when the physician reported no disease activity (PhysGA = 0), 45.2% of the children still reported some physical disability. For both patient- and physician-reported outcome measures, regression analysis showed associations with physical disability (CHAQ > 0) (Table 5), with the strongest associations between patient-reported outcome measures and physical disability. Children reporting some disease-related pain had higher odds of reporting physical disability (adjusted OR 14.1, 95% CI 6.8, 29.2) compared to those reporting no pain. Similarly, children reporting that their lives were somewhat negatively affected by their disease (PatGA > 0) had higher odds of reporting physical disability (adjusted OR 14.0, 95% CI 6.2, 31.6) compared to those reporting no disease-related impact on well-being. We also found a strong association between patient-reported poor physical health using the generic instrument (CHQ PhS < 40) and physical disability using the disease-specific instrument (CHAQ > 0). Participants reporting poor physical health (CHQ PhS < 40) had much higher odds of also reporting physical disability (CHAQ > 0), compared to those reporting a physical health within the normal range (adjusted OR 19.0, 95% CI 5.6, 64.1). Similarly, those reporting poor mental health (CHQ PsS < 40) also had higher odds of reporting physical disability compared to those with normal mental health (adjusted OR 8.1, 95% CI 1.8, 37.3).

**Table 5:** Self-reported physical disability in the JIA group according to other outcome measures

		**CHAQ score**		**Poor Physical health**	
		*** = 0***	***> 0***	***CHAQ score > 0***	
**Other outcome measures**	***N***^***a***^	***n (%)***	***n (%)***	***OR (95% CI) crude***	***OR (95% CI) adjusted***^***b***^
VAS pain^c^					
= 0	82	63 (76.8)	19 (23.2)	1.0 (ref.)	1.0 (ref.)
> 0	139	30 (21.6)	109 (78.4)	12.0 (6.3, 23.1)	14.1 (6.8, 29.2)
PatGA VAS^d^					
= 0	61	50 (82.0)	11 (18.0)	1.0 (ref.)	1.0 (ref.)
> 0	160	43 (26.9)	117 (73.1)	12.4 (5.9, 25.9)	14.0 (6.2, 31.6)
Morning stiffness					
< 15 min	166	85 (51.2)	81 (48.8)	1.0 (ref.)	1.0 (ref.)
≥ 15 min	49	7 (14.3)	42 (85.7)	6.3 (2.7, 14.8)	7.0 (2.9, 16.9)
CHQ PhS					
≥ 40	165	89 (53.9)	76 (46.1)	1.0 (ref.)	1.0 (ref.)
< 40	53	3 (5.7)	50 (94.3)	19.5 (5.9, 65.1)	19.0 (5.6, 64.1)
CHQ PsS					
≥ 40	199	90 (45.2)	109 (54.8)	1.0 (ref.)	1.0 (ref.)
< 40	19	2 (10.5)	17 (89.5)	7.0 (1.6, 31.2)	8.1 (1.8, 37.3)
PhysGA^e^					
= 0	146	80 (54.8)	66 (45.2)	1.0 (ref.)	1.0 (ref.)
> 0	75	13 (17.3)	62 (82.7)	5.8 (2.9, 11.4)	6.2 (3.1, 12.6)
Disease status^f^					
Rem. off med.	29	25 (86.2)	4 (13.8)	1.0 (ref.)	1.0 (ref.)
Inactive	106	55 (51.9)	51 (48.1)	5.8 (1.9, 17.8)	5.4 (1.7, 17.3)
Active	86	13 (15.1)	73 (84.9)	35.1 (10.5, 117.6)	36.3 (10.3, 128.1)
Medication ongoing^g^					
No DMARDs	77	43 (55.8)	34 (44.2)	1.0 (ref)	1.0 (ref)
DMARDs	144	50 (34.7)	94 (65.3)	2.4 (1.4, 4.2)	2.2 (1.2, 3.9)
Medication ever^g^					
No DMARDs	55	30 (54.5)	25 (45.5)	1.0 (ref)	1.0 (ref)
DMARDs	166	63 (38.0)	103 (62.0)	2.0 (1.1, 3.6)	1.9 (1, 3.6)
JIA category^h^					
Oligo. persistent	76	40 (52.6)	36 (47.4)	1.0 (ref)	1.0 (ref)
Other	145	53 (36.6)	92 (63.5)	1.9 (1.1, 3.4)	2.0 (1.1, 3.6)

*JIA* Juvenile idiopathic arthritis, *N* Number, *CHAQ* Childhood Health Assessment Questionnaire, *OR* Odds ratio, *CI* Confidence interval, *VAS* Visual analogue scale, *ref*. reference, *PatGA* Patient Global Assessment of disease impact on well-being, *CHQ* Child Health Questionnaire, *PhS* Physical Summary Score, *PsS* Psychosocial Summary Score, *PhysGA* Physician's global assessment of disease activity, *Rem. off med.* Remission off medication, *DMARDs* disease modifying anti-rheumatic drugs, including synthetic (methotrexate, hydroxychloroquine, cyclosporine, mycophenolate mofetil) and/or biologic DMARDs (etanercept, infliximab, adalimumab, tocilizumab, abatacept, certolizumab, golimumab, rituximab). *Oligo.* Oligoarticular

^a^N assessed for each variable, excluding missing/unknown values. Total participants, *N* = 221

^b^Adjusted for sex, age, disease duration, and Iso-BMI

^c^Self-reported disease-related pain measured on a 21-numbered circle VAS pain (0 = no pain, 10 = maximum pain)

^d^Assessed by the patient/parent on a 21-numbered circle VAS (0 = very well, 10 = very poor)

^e^Assessed by the physician on a 21-numbered circle VAS (0 = inactive, 10 = maximal activity)

^f^Disease status according to [14] and [15]: Rem. off med. for ≥ 12 months. Inactive disease = inactive disease on medication < 6 months or off medication < 12 months, or remission on medication (inactive disease on medication for > 6 months). Active disease = flare or continuous active disease

^g^Ongoing refers to ongoing medication for arthritis or uveitis at the study visit. Ever refers to medication ever used for arthritis or uveitis during the disease course, including ongoing medication. Registered medication was dichotomized into No DMARDs and DMARDs

^h^Categories defined according to the International League of Association for Rheumatology (ILAR) classification criteria and dichotomized into oligoarticular persistent JIA versus all other categories

Compared to the patient-reported outcome measures, the associations were somewhat weaker for the physician-reported measures. Participants with JIA assessed by the physicians to have some degree of disease activity had higher odds of self-reported physical disability (adjusted OR 6.2, 95% CI 3.1, 12.6). An association could also be seen between disease status and physical disability. Compared to participants in remission off medication, the odds of self-reported physical disability were higher among participants with inactive disease (adjusted OR 5.4, 95% CI 1.7, 17.3), and substantially higher among participants with active disease (adjusted OR 36.3, 95% CI 10.3, 128.1). Some associations were also seen between treatment with DMARDs or JIA categories other than oligorticular JIA, and physical disability.

## Discussion

A majority (57.9%) of children with JIA reported some physical disability scored with the disease-specific instrument CHAQ. Children with JIA reported significantly poorer physical health (CHQ PhS), and also a minor reduction in psychosocial health (CHQ PsS) scored with the generic instrument CHQ, compared to sex- and age-matched controls. Strong associations were found between patient-reported outcome measures, such as pain and global wellbeing, and patient-reported physical disability (CHAQ > 0) in JIA. Participants reporting a physical health below the norm (CHQ PhS < 40) had much higher odds of also reporting physical disability (CHAQ > 0). There was also a clear association between physician-reported disease activity and patient-reported physical disability, as well as between disease status, especially active disease, and physical disability.

Our study is based on the NorJIA study, which included a large number of participants with JIA in all age groups from 4 to 16 years, in addition to a matched control cohort. Furthermore, all JIA categories were represented in the study population. Another strength is that the participants were recruited from three different locations, representing three different parts of the country. The organization of the Norwegian public health system, with free clinical consultations for children, equal access to a common public health service with few private actors, and a regional responsibility to pediatric rheumatology ensuring that all children with JIA are seen by pediatric rheumatologists at the university hospitals, reduces the risk of selection bias. Also, a stable and relatively homogenous population makes comparisons both with the controls and within the JIA group, more reliable. Validated instruments were used for both patient- and physician-reported outcomes, and clinical examinations were performed by experienced pediatric rheumatologists following a standardized study protocol, further strengthening the study.

Nevertheless, several limitations must be taken into consideration. Even with a relatively high response rate of 63%, possible selection bias must be considered. The mean age was 1.5 years lower among those who declined participation than in the study population. Possible explanations for this may be that parents of the youngest children with JIA were more hesitant of letting their child participate in a study consisting of two days of clinical examinations which might be challenging for the youngest children. However, no clear association could be seen between age at clinical examination and self-reported physical disability (CHAQ > 0). Furthermore, we cannot exclude a selection bias towards recruiting more children with active disease. Children in remission are less often seen at the hospital and might also be more hesitant to participate in an extensive study. Another limitation is that some of the PROMs are reported by the parents and others by the child itself. As the parents experience the disease of the child from a different perspective than the child, this is a known challenge when making comparisons between PROM scores [21, 22]. In addition, we cannot exclude potential impact of dependent measurement error related to participants answering multiple self-reported outcome measures. Furthermore, many children scored zero on CHAQ, and we cannot exclude that some of these children felt quite well, but not without any disability, and were grouped together with children with full ability, due to the floor effect of the instrument [23, 24]. Finally, comparison between the JIA categories was not feasible, due to low number of children in some categories.

The importance of patient-reported outcomes is highly recognized in JIA research and also for use in clinical settings [8–10, 25]. Several studies have developed and evaluated PROMS for use in research and clinical settings, but to our knowledge there are few studies on the association between the different PROMs, and between PROMs and physician-reported outcome measures. Compared to our results, showing that a majority of the children scored > 0 on the CHAQ (58%), other studies have generally found lower percentages of children with disability. In several studies approximately half of the participants reported > 0 on the CHAQ or on the adult version – the Health Assessment Questionnaire (HAQ) [23, 26–28], somewhat lower than our results. Three Nordic long-term follow-up studies showed markedly lower numbers; Flatø et al. found 36% of participants reporting HAQ > 0 at 14.9 years after disease onset [29], Nordal et al. and Glerup et al. found 32% and 28% of participants reporting CHAQ or HAQ > 0 at eight and 18 years after disease onset, respectively [5, 30].

Comparison between studies is always hampered by different study design and different study populations. The most apparent explanation for our physical disability numbers being higher than in the other studies is that our participants were generally younger, with varying disease duration and thus many with more recent JIA diagnoses [26–30]. One study in which participants had a median JIA duration of only 11 months reported a mean CHAQ score of 0.5, notably higher than our mean of 0.3 [31]. During the first years of disease activity, more patients will have active disease or flares until adequate medication is found. Furthermore, patients with a shorter disease duration could be more prone to focus on the symptoms, as they are not so familiar with their disease. A Norwegian qualitative study from 2009 indicated that participants gradually adjusted psychosocially, finding coping mechanisms and adapted their perspectives to tackle the disease burden that JIA entails [32]. This is consistent with the small negative association we found between disease duration and physical disability. A follow-up study of this cohort might have demonstrated if the proportion of participants reporting disability was reduced with time, but a follow-up was beyond the scope of this study. That being said, neither we nor Minden et al. found a substantial relationship between disease duration and physical disability (CHAQ or HAQ) [33]. Interestingly, Zak and Pedersen found a positive correlation between HAQ score and disease duration [27]. Another apparent difference between our study and the other studies was the proportion of patients in remission off medication. In our study, only 13% of the participants were in remission off medication, whereas the number ranged from 33 to 63% in the long-term studies. This supports the impression that our study group had more active disease [5, 26–30, 33]. As for the other outcome measures, the proportion of participants with CHQ physical < 40 was 24% in the Nordic study [30] versus 19% in our study. The equivalent proportion for CHQ psychosocial < 40 was 8.7 in both our and Nordal’s participants [30]. Furthermore, our results showed that the pain level was strongly associated with physical disability. Similarly, Minden et al. found that pain scores in young adults with JIA had a statistically significant correlation with HAQ scores [33]. We also found an association between disease impact on global well-being reported by the patient (PatGA) and physical disability comparable to the result found by Minden et al. in 2002 [33].

Our results also showed a strong association between the physician-reported global assessment (PhysGA) of disease activity and patient-reported physical disability (CHAQ). Other studies have also found association between assessment by physicians and self-reported physical functioning: Both Zak and Pedersen, and Minden et al. found that Steinbrocker functional classes correlated with HAQ [27, 33]. Craig et al. on the other hand, found little association between PhysGA and CHAQ [23]. Several long-term follow-up studies have found associations between disease activity and increased HAQ score [27, 28, 33]. This strengthens our results demonstrating that active disease had the strongest association to physical disability of all the variables we studied (adjusted OR 36.3, 95%, CI 10.3, 128.1). It also strengthens the impression that disease activity, and perhaps not so much permanent damage, accounts for a large part of the patient’s evaluation of their disease-related physical disability.

The results of this study clearly support the notion that patient-reported physical health and disability are important supplements to the physician’s evaluation of the patient, providing a more complete picture of the disease impact on the patient [9, 10]. Despite being a subjective measure, there is an evident association between other outcome instruments and the CHAQ, including both other patient-reported tools, and more objective clinical activity measures. When aiming to provide individualized therapy and achieve inactive disease and remission, the traditional physician-assessed clinical measures are insufficient. More details about the patient’s view are needed. The use of PROMs such as CHAQ is a validated, systematic method for collecting such information, and is therefore highly relevant both in a research setting, and as part of the routine clinical follow-up.

## Conclusions

Our results demonstrated that patient-reported physical health in JIA remains low and much lower than in children without JIA, and that there was a strong association between other patient-reported outcome measures and patient-reported physical disability. There was also a strong association between the physicians’ assessment of disease activity and patient-reported physical disability. Our results support the importance of PROMs in the follow-up of JIA patients. PROMs such as CHAQ can provide reliable information from the patient’s perspective which the physician can use to get the full picture of the disease. Ultimately, we believe that bringing forth the patient’s voice can result in both a more holistic and individualized patient care.

## Supplementary Information

Additional file 1: Supplemental Table 1. Self-reported physical and psychosocial health in participants with juvenile idiopathic arthritis (JIA) and controls according to sex and age.

Additional file 2: Supplemental Fig. 1. Distribution of The Childhood Health Assessment Questionnaire (CHAQ) scores (range 0–3, 0 = no disability, 3 = maximum disability) reported by the children in the juvenile idiopathic arthritis (JIA) group (*n* = 221), according to sex.

## Data Availability

The NorJIA study are described on https://norjia.com/ and https://ClinicalTrials.gov/show/NCT03904459. The dataset used in this study is not publicly available but can be made available at reasonable request to the corresponding author.

## Abbreviations

ACR: American College of Rheumatology
ANA: Anti-nuclear antibody
bDMARDs: Biologic disease-modifying antirheumatic drugs
BMI: Body mass index
CHAQ: Child Health Assessment Questionnaire
CHQ: Child Health Questionnaire
CHQ-PF50: Child Health Questionnaire Parent form
CHQ PhS: Physical Summary score
CHQ PsS: Psychosocial Summary score
CI: Confidence interval
CRP: C-reactive protein
DMARDs: Disease-modifying antirheumatic drugs
ESR: Erythrocyte sedimentation rate
GDPR: General Data Protection Regulation
HAQ: Health Assessment Questionnaire
HLA-B27: Human leukocyte antigen B27
ILAR: International League of Associations for Rheumatology
IOTF: International Obesity Task Force
IQR: Interquartile range
ISM: Department of Public Health and Nursing
JIA: Juvenile idiopathic arthritis
NorJIA: Norwegian study on imaging, oral health and quality of life in children with JIA
NSAIDs: Nonsteroidal anti-inflammatory drugs
OR: Odds ratio
PatGA: Patient’s global assessment of overall well-being
PhysGA: Physician’s global assessment of disease activity
PRINTO: The Paediatric Rheumatology INternational Trials Organisation
PROM: Patient-reported outcome measure
REC: Regional Committees for Medical Research Ethics
RF: Rheumatoid factor
SD: Standard deviation
sDMARDs: Synthetic disease-modifying antirheumatic drugs
VAS: Visual analogue scale
