# Supplementary material for: The patient’s voice: a cross-sectional study of physical health and disability in juvenile idiopathic arthritis
Source: Pediatr Rheumatol Online J. 2024 Nov 18;22:100. doi: 10.1186/s12969-024-01034-7 (PMC11572323; doi:10.1186/s12969-024-01034-7)
Supplement: Supplementary file 1 — Additional file 1: Supplemental Table 1. Self-reported physical and psychosocial health in participants with juvenile idiopathic arthritis (JIA) and controls according to sex and age. [file 12969_2024_1034_MOESM1_ESM.docx]

**Supplemental Table 1: Self-reported physical and psychosocial health in participants with juvenile idiopathic arthritis (JIA) and controls according to sex and age**

|  | **JIA** | | | | |  | **Controls** | | | | |
| --- | --- | --- | --- | --- | --- | --- | --- | --- | --- | --- | --- |
|  | **Sex** | |  | **Age** | |  | **Sex** | |  | **Age** | |
|  | ***F*** | ***M*** |  | ***<9 y*** | ***≥9 y*** |  | ***F*** | ***M*** |  | ***<9 y*** | ***≥9 y*** |
| **Mean Physical Summary Score (CHQ PhS)** | | | | | | | | | | | |
| N^a^ | 129 | 89 |  | 45 | 173 |  | 123 | 84 |  | 42 | 165 |
| Mean  (SD) | 45.1 (11.1) | 46.5 (10.8) |  | 47.8 (10.1) | 45.1 (11.2) |  | 56.0 (3.7) | 55.9 (4.5) |  | 57.2 (3.0) | 55.7 (4.2) |
| n <40  (%) | 31 (24.0) | 22 (24.7) |  | 9  (20.0) | 44 (25.4) |  | 1  (0.8) | 0  (0.0) |  | 0  (0.0) | 1  (0.6) |
| **Mean Psychosocial Summary Score (CHQ PsS)** | | | | | | | | | | | |
| N^a^ | 129 | 89 |  | 45 | 173 |  | 123 | 84 |  | 42 | 165 |
| Mean  (SD) | 53.2 (7.7) | 52.4 (8.6) |  | 53.2 (7.6) | 52.8 (8.2) |  | 55.6 (6.2) | 55.1 (6.8) |  | 55.0 (4.7) | 55.5 (6.8) |
| n <40  (%) | 9 (7.0) | 10 (11.2) |  | 3  (6.7) | 16  (9.2) |  | 2  (1.6) | 2  (2.4) |  | 0  (0.0) | 4  (2.4) |

*JIA = Juvenile idiopathic arthritis, F = Female, M = Male, y = years, CHQ = Child Health Questionnaire as assessed by the 50-item Child Health Questionnaire-Parent Version (CHQ-PF50), PhS = Physical Summary Score, N = Number, SD = Standard deviation, PsS = Psychosocial Summary Score*

^a^ *Number of participants assessed for each variable, excluding missing/unknown values. Total participants, N = 218, excluding 3 participants with missing CHQ (2 females, 1 male). Total controls, N = 207, excluding 17 participants with missing CHQ (11 females, 6 males).*
