# Supplementary figures and images for: The patient’s voice: a cross-sectional study of physical health and disability in juvenile idiopathic arthritis
Source: Pediatr Rheumatol Online J. 2024 Nov 18;22:100. doi: 10.1186/s12969-024-01034-7 (PMC11572323; doi:10.1186/s12969-024-01034-7)

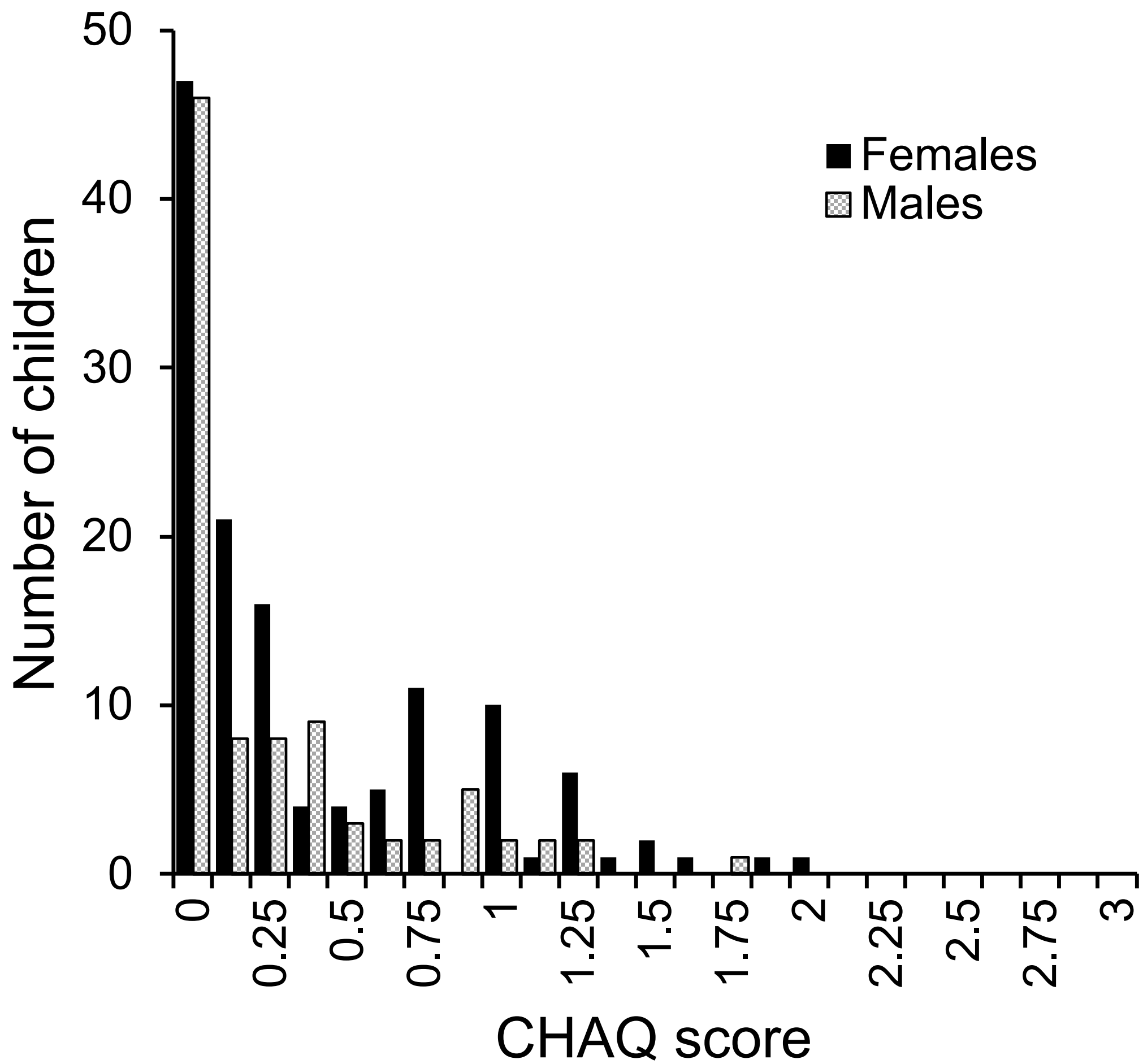

Supplement: Supplementary file 2 — Additional file 2: Supplemental Fig. 1. Distribution of The Childhood Health Assessment Questionnaire (CHAQ) scores (range 0–3, 0 = no disability, 3 = maximum disability) reported by the children in the juvenile idiopathic arthritis (JIA) group (n = 221), according to sex. [file 12969_2024_1034_MOESM2_ESM.pdf]
